# Transcriptome sequencing of circular RNA reveals the involvement of hsa‐SCMH1_0001 in the pathogenesis of Parkinson's disease

**DOI:** 10.1111/cns.14435

**Published:** 2023-09-04

**Authors:** Qiao Wang, Huizhi Wang, Xuemin Zhao, Chunlei Han, Chong Liu, Zhibao Li, Tingting Du, Yunpeng Sui, Xin Zhang, Jianguo Zhang, Yilei Xiao, Guoen Cai, Fangang Meng

**Affiliations:** ^1^ Department of Functional Neurosurgery, Beijing Neurosurgical Institute Capital Medical University Beijing China; ^2^ Beijing Key Laboratory of Neurostimulation Beijing China; ^3^ National Center of Gerontology, Institute of Geriatric Medicine, Chinese Academy of Medical Sciences Beijing Hospital Beijing China; ^4^ Department of Neurophysiology, Beijing Neurosurgical Institute Capital Medical University Beijing China; ^5^ Department of Neurosurgery, Beijing Tiantan Hospital Capital Medical University Beijing China; ^6^ Department of Neurosurgery Liaocheng People's Hospital Liaocheng China; ^7^ Department of Neurology Fujian Medical University Union Hospital Fuzhou China; ^8^ Fujian Key Laboratory of Molecular Neurology, Institute of Clinical Neurology, Institute of Neuroscience Fujian Medical University Fuzhou China; ^9^ Chinese Institute for Brain Research Beijing China

**Keywords:** circular RNA, exosomes, next‐generation sequencing, Parkinson's disease

## Abstract

**Background:**

Parkinson's disease (PD) is the second most common neurodegenerative disease. Exosomes are endosome‐derived extracellular vesicles that can take part in intercellular communication. Circular RNAs (circRNAs) are noncoding RNAs characterized by covalently closed‐loop structures, which perform a crucial function in many diseases.

**Aim:**

To clarify the expression and function of exosomal circRNSs of PD patients and look for circRNAs that might be related to the pathogenesis of PD.

**Materials and Methods:**

We examined circRNA and mRNA expression profiles in peripheral exosomes from PD patients (*n* = 23) and healthy controls (*n* = 15) using next‐generation sequencing (NGS) technology, functional annotation, and quantitative polymerase chain reaction. Correlation analysis was performed between the expression levels of the circRNAs and the clinical characteristics of PD patients. The binding miRNAs and target genes were predicted using TargetScanHuman, miRDB, and miRTarBase. The predicted target genes were compared with the differentially expressed mRNAs in sequencing results.

**Results:**

According to the NGS, 62 upregulated and 37 downregulated circRNAs in the PD group were screened out. Correlation analysis revealed that hsa‐SCMH1_0001 has strong clinical relevance. We identified 17 potential binding miRNAs of hsa‐SCMH1_0001 with 149 potential target genes. ARID1A and C1orf115 belong to the intersection of the predicted target genes and the differentially expressed mRNAs obtained by sequencing.

**Conclusion:**

This study suggested that hsa‐SCMH1_0001 and its target genes ARID1A and C1orf115 are downregulated in PD patients and may be involved in the occurrence of PD.

## Graphical abstract

CircRNA expression profiles were examined in peripheral exosomes from PD patients and healthy controls using next‐generation sequencing. Ninety‐nine DEcircRNAs were screened out and three of them were selected for quantitative polymerase chain reaction. Correlation analysis revealed that hsa‐SCMH1_0001 has strong clinical relevance. Seventeen binding miRNAs of hsa‐SCMH1_0001 and 149 target genes including ARID1A and C1orf115 were predicted using multiple databases.

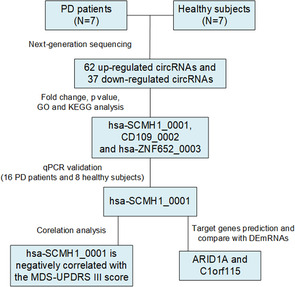

## 1 INTRODUCTION

Parkinson's disease (PD) is a common neurodegenerative disease that is generally not diagnosed until years after its initial onset. PD is characterized by the loss of dopaminergic neurons in the substantia nigra pars compacta and the formation of Lewy bodies arising from misfolded α‐synuclein.^1^, ^2^ However, the precise mechanisms underlying PD remain unclear. The diagnosis and treatment of PD are as complex as its symptoms. Many biomarkers are currently under investigation for the early diagnosis of PD, including genetic biomarkers.^3^, ^4^, ^5^

Exosomes are endosome‐derived extracellular vesicles, which range in size from 40 to 160 nm. Exosomes are composed of a lipid bilayer that contains proteins, lipids, DNA, and RNA, which are involved in intercellular communication.^6^ They can be detected in blood, saliva, urine, and cerebrospinal fluid. To date, studies have shown that exosomes are associated with immune responses, viral pathogenicity, cardiovascular disease, central nervous system diseases, and cancer.^7^, ^8^ Because exosomes can cross the blood–brain barrier (BBB) directly, exosomes released by neurons, oligodendrocytes, microglia, and astrocytes can be detected in the peripheral blood.^9^ Previous studies have shown that α‐synuclein levels were elevated in PD exosomes in the serum, and that pathogenic forms of α‐synuclein may spread via exosomes.^10^ Similarly, the PD‐related mutant LRRK2 was shown to be released in exosomes.^11^ Sang et al.^12^ demonstrated that circSNCA served as a miR‐7 sponge resulting in the upregulation of SNCA in PD while downregulation of circSNCA could decrease apoptosis and induce autophagy.

Circular RNAs (circRNAs) are an endogenous class of noncoding RNAs characterized by covalently closed‐loop structures. CircRNAs are insensitive to nucleases due to their circular structure, which makes them more stable than linear RNAs. They are enriched in the nervous system and have well‐conserved sequences.^13^ CircRNAs have complex tissue‐ and stage‐specific expression patterns. Because circRNAs are stable in the blood and extracellular vesicles, they could be potential biomarkers for disease. Indeed, circRNAs have been proposed as biomarkers for many diseases, such as lupus erythematosus, tuberculosis, rheumatoid arthritis, diabetes, cancer, and major depressive disorder.^14^, ^15^, ^16^, ^17^, ^18^, ^19^ In addition, the expression of circRNAs has been linked to neurological diseases, including neuropsychiatric disease, neurodegenerative disease, and brain tumors.^20^, ^21^, ^22^ For example, Hanson et al.^23^ speculated that circRNAs act as sponges for miR‐7 and may be crucial factors in PD while Wang et al.^24^ identified a circRNA‐associated‐ceRNA network in an Alzheimer's disease rat model using microarray analysis. IQCK, MAP4K3, EFCAB11, DTNA, and MCTP1 were found to be overexpressed in multiple system atrophy brains,^25^ and amyotrophic lateral sclerosis‐associated fused in sarcoma (FUS) mutation was shown to lead to circRNA production.^26^ Although, taken together, these studies suggested that both circRNAs and exosomes may play a significant role in the pathogenesis of PD, the expression pattern of circRNAs in peripheral blood exosomes of PD patients requires further investigation.

Here, we screened circRNAs in peripheral exosomes from PD patients, and identified hsa‐CD109_0002, hsa‐SCMH1_0001, and hsa‐ZNF652_0003 as differentially expressed circRNAs (DEcircRNAs) in PD patients compared with healthy controls. Furthermore, we demonstrated that the expression level of hsa‐SCMH1_0001 was consistent with the Movement Disorder Society (MDS)‐sponsored Unified Parkinson's Disease Rating Scale part III (MDS‐UPDRS III) score of PD patients. Analysis of competing endogenous RNAs (ceRNAs) revealed key genes that were regulated by hsa‐SCMH1_0001 and may be involved in the pathological changes associated with PD.

## 2 METHOD

### 2.1 Patients

Twenty‐three PD patients form Beijing Tiantan Hospital and 15 healthy volunteers were included in this study. All participants were informed about the purpose and risk of the study and subsequently gave their written informed consent. All subjects underwent evaluations including medical history, physical examination, laboratory tests, and neuropsychological assessment. PD patients were diagnosed by movement disorder specialists in accordance with the British Brain Bank Criteria and were stringently selected to exclude other major diseases. Next‐generation sequencing (NGS) was performed on seven PD cases and seven healthy controls whose age and sex were matched. Real‐time PCR was carried out on samples from the remaining 16 PD cases and eight healthy controls. This research was approved by the local medical ethics committee (Beijing Tiantan Hospital, Capital Medical University, Beijing, China; reference number KYSQ 2019‐103‐01).

Blood samples (6 mL per sample) were collected in ethylenediaminetetraacetic acid tubes following puncture of the median cubital vein. The plasma was aspirated after centrifugation at 3000 × *g* for 15 min at 4°C and stored at −80°C. Samples were thawed prior to exosome isolation. Exosomes were purified using the ultracentrifugation method. Briefly, samples were centrifuged at a low speed of 300 × *g* for 15 min to discard cell debris. The supernatant was diluted using a sevenfold volume of phosphate‐buffered saline (PBS) and purified by ultracentrifugation at 13,000 × *g* for 30 min. Large particles were discarded through a 0.22 μm filter, and the supernatant was ultracentrifuged using a P50A72‐986 rotor (CP100NX; Hitachi) at 100,000 × *g* for 2 h to pellet the exosomes. The pellet was resuspended in PBS and underwent further centrifugation at 100,000 × *g* for 2 h. All procedures were performed at 4°C. The exosomes were resuspended in PBS and stored at −80°C for further use.

Transmission election microscopy (TEM), nanoparticle tracking analysis (NTA), and western blotting were used for identification of the exosomes as previously described.^27^ The antibodies used were CD9‐antibody (60232‐1, Proteintech, 1:1000, 23‐27KD), Tsg101‐antibody (ab125011, abcam, 1:1000, 45KD), HSP70‐antibody (ab181606, abcam, 1:1000, 70KD), and calnexin‐antibody (10427‐2, Protentech, 1:500, 90KD).

### 2.2 Exosomal RNA isolation

Exosome samples were thawed at 4°C and centrifuged at 12,000 × *g* for 10 min to discard impurities. The supernatant was transferred to a 1.5 mL centrifuge tube, mixed with 750 μL of TRIzol LS Reagent (Life Technologies Corp.) and 20 μL of glacial acetic acid, and then left at room temperature for 3–5 min until phase separation was observed. The yellow organic phase, interlayer, and colorless aqueous phase were separated by centrifugation at 10,000 × *g* for 10 min at 4°C. The aqueous phase that contained RNA was aspirated carefully to another tube. An equal volume of cold isopropyl alcohol was added to the supernatant and incubated at room temperature for 10–20 min. The supernatant was centrifuged at 4°C at 10,000 × *g* for 10 min. Then, 75% ethanol (prepared with RNase‐free water, 1 mL) was added to the RNA precipitate, centrifuged at 1800‐4500 × *g* at 4°C for 1–2 min, then the supernatant was discarded. RNase‐free water (50–100 μL) was added to the precipitate, gently mixed to dissolve the RNA, and stored at −70°C. RNA integrity was measured using denatured agarose gel electrophoresis (Sangon Bioengineering Co., Ltd., A500016‐0250). The concentration of purified RNA was determined by Nanodrop ND‐1000 (USA, Thermo).

The Ribo‐Zero rRNA Removal Kit (Illumina, MRZG12324) was used to remove ribosomal RNA. RNA preconditioning was performed with a TruSeq Total RNA Library Prep Kit (Illumina, 20020596) according to the manufacturer's instructions. One hundred and fifty to three hundred base pairs of DNA were purified by nucleic acid electrophoresis (Qiaquick Gel Extraction Kit, Qiagen, 28704). Library size and concentration were measured using Agilent 2100 Analyzer (Agilent). The 10 pM library was denatured into single‐stranded DNA molecules, which were captured on Illumina flow cell (Illumina) and amplified into clusters in situ. Exosome isolation and sequencing were provided by Echo Biotech Co., Ltd.

### 2.3 Expression analysis of circRNAs and mRNAs

The analysis of DEcircRNAs and differentially expressed mRNAs (DEmRNAs) between PD patients and healthy controls was based on sequencing results obtained using the Illumina HiSeq2500 system. Raw reads were stored in the FASTQ format and the *Q*‐score was calculated using the program Phred with *Q*_‐score_ = −10 × log_10_(p) representing the probability of base calling error.^28^ The raw data were processed to remove the readouts containing adapter and low‐quality data. An independent Burrows–Wheeler Aligner (BWA) alignment to reference RNA sequences was performed to obtain the mapped data. CIRI software was used to predict circRNAs, the results were mapped to the circBase database (http://www.circbase.org/), and known or newly predicted circRNAs were detected. The expression data of circRNAs were normalized using the TPM (transcripts per million) method,^29^ and the mRNA expression level was normalized using the FPKM (fragments per kb of transcript per million fragments mapped) method.^30^

DEcircRNAs between PD patients and healthy controls were generated using the DESeq^31^ method with fold changes ≥1.5 and *p* < 0.05 set as the threshold. EdgeR was used to analyze the differential expression of mRNA using the same threshold parameters.^32^

### 2.4 Functional annotation and enrichment analysis

Functional annotation was performed on the identified DEcircRNAs and DEmRNAs. Gene Ontology (GO) analysis was carried out using the GO seq R packages based on a Wallenius noncentral hyper‐geometric distribution. The GO term enrichment was performed using clusterProfiler with the selection criteria of *p* < 0.05.^33^ The statistical enrichment in the Kyoto Encyclopedia of Genes and Genomes (KEGG) pathway was tested by KOBAS software.^27^ Clusters of orthologous groups of proteins (COG) were also used for orthologous classification of gene products.^34^ The functional annotation of DEmRNAs has been described previously.^35^

### 2.5 Quantitative polymerase chain reaction validation

Total RNA was extracted from exosomal samples. The quantification and quality of the total RNA were assessed by NanoDrop ND‐1000 spectrophotometer (Thermo Fisher). The RNA was reverse transcribed into complementary DNA using the Gene Amp PCR System 9700 (Applied Biosystems) according to the manufacturer's instructions. Based on the raw data, fold change, and *p*‐value, hsa‐CD109_0002, hsa‐SCMH1_0001, and hsa‐ZNF652_0003 were selected for real‐time quantitative polymerase chain reaction (qPCR) using the QuantStudio 5 Real‐time PCR System (Thermo Fisher) with three replication each. GAPDH was used as a housekeeping gene. Data were calculated as relative expressions according to the 2^‐ΔΔCT^ principle. The primer sequences were 5′‐TCA GCT ATC TTC CCA TCC AA‐3′ and 5′‐CTG GGT ACG TCC GGT TAC AC‐3′ for hsa‐CD109_0002; 5′‐TCG GAG GCA GGA TAG GGA TT‐3′ and 5′‐CTC CTG AAC CGG ATA CCA GC‐3′ for hsa‐SCMH1_0001; and 5′‐GGA GTG CAC CTT GAG TGA CA‐3′ and 5′‐AGC AGT ACT TCG ACG AAC ACA‐3′ for hsa‐ZNF652_0003.

### 2.6 The ceRNA analysis

The circRNAs–miRNA–mRNA ceRNA network was constructed as follows: Functionally, circRNAs can act as miRNA sponges to regulate the expression level of disease‐related miRNAs. The binding sites of circRNAs and miRNAs were predicted using CircInteractome (www.circinteractome.nia.nih.gov).^36^ The target genes of miRNAs were predicted via the TargetScanHuman (version 7.1; www.targetscan.org),^37^ miRDB (http://www.mirdb.org/),^38^ and miRTarBase (http://mirtarbase.mbc.nctu.edu.tw/php/index.php) databases.^39^ The intersection of target genes with total context++ score ≤−0.2 in the TargetScanHuman database, target genes with a target score ≥80 in the miRDB database, and target genes in the miRTarBase database were selected as the final target genes. The circRNA–miRNA–mRNA network was constructed and visualized using Cytoscape software (version 3.8.2; www.cytoscape.org).^40^

### 2.7 Statistical analysis

Nonparametric data are displayed as median and interquartile range while parametric data are presented as mean ± SD. All statistical analyses were carried out using SPSS statistical software version 25.0 (SPSS) and GraphPad Prism version 9.0 (GraphPad Software). Normality test was conducted using the Shapiro–Wilk's test, and variance homogeneity test was conducted using the Levene's test. Expression levels of circRNAs were compared using Mann–Whitney test because the qPCR data does not fit the Gaussian distribution. The data of age and course of disease were Gaussian distribution and were compared using Student's *t*‐test. The Pearson's chi‐squared test was used to compare gender composition between the two groups. The Spearman's rank correlation test was used for statistical analysis of the circRNAs expression level and clinical characteristics. *p* < 0.05 was considered to be statistically significant.

## 3 RESULTS

### 3.1 Clinical characteristics of PD patients and identification of exosomes

The clinical characteristics of PD patients are listed in Tables 1 and 2, and Table S1. Samples from seven PD patients and seven healthy controls were used for NGS while 16 PD patient and eight healthy control samples were used for qPCR. No significant differences in age and sex were observed between the PD and control groups for both analyses. Two experienced physicians specializing in PD assessed the condition of all patients, focusing on the Hoehn and Yahr stage and the MDS‐UPDRS III score. None of the patients had relatives with Parkinson's disease.

**TABLE 1:** Demographic and clinical characteristics of next‐generation sequencing participants.

Group	PD (*n* = 7)	HC (*n* = 7)	*p*‐value
Age, mean ± SD	61.43 ± 10.00	55.29 ± 4.96	0.18
Sex, females/males	0.75	0.75	>0.99
Disease duration, mean ± SD	8.29 ± 3.49		
H&Y score	≥3		

**TABLE 2:** Demographic and clinical characteristics of real‐time quantitative PCR participants.

Group	PD (*n* = 16)	HC (*n* = 8)	*p*‐value
Age, mean ± SD	64.13 ± 8.37	58.63 ± 10.56	0.18
Sex, females/males	0.78	1	0.77
Disease duration, mean ± SD	9.69 ± 4.01		
H&Y score	≥3		

**FIGURE 1:**
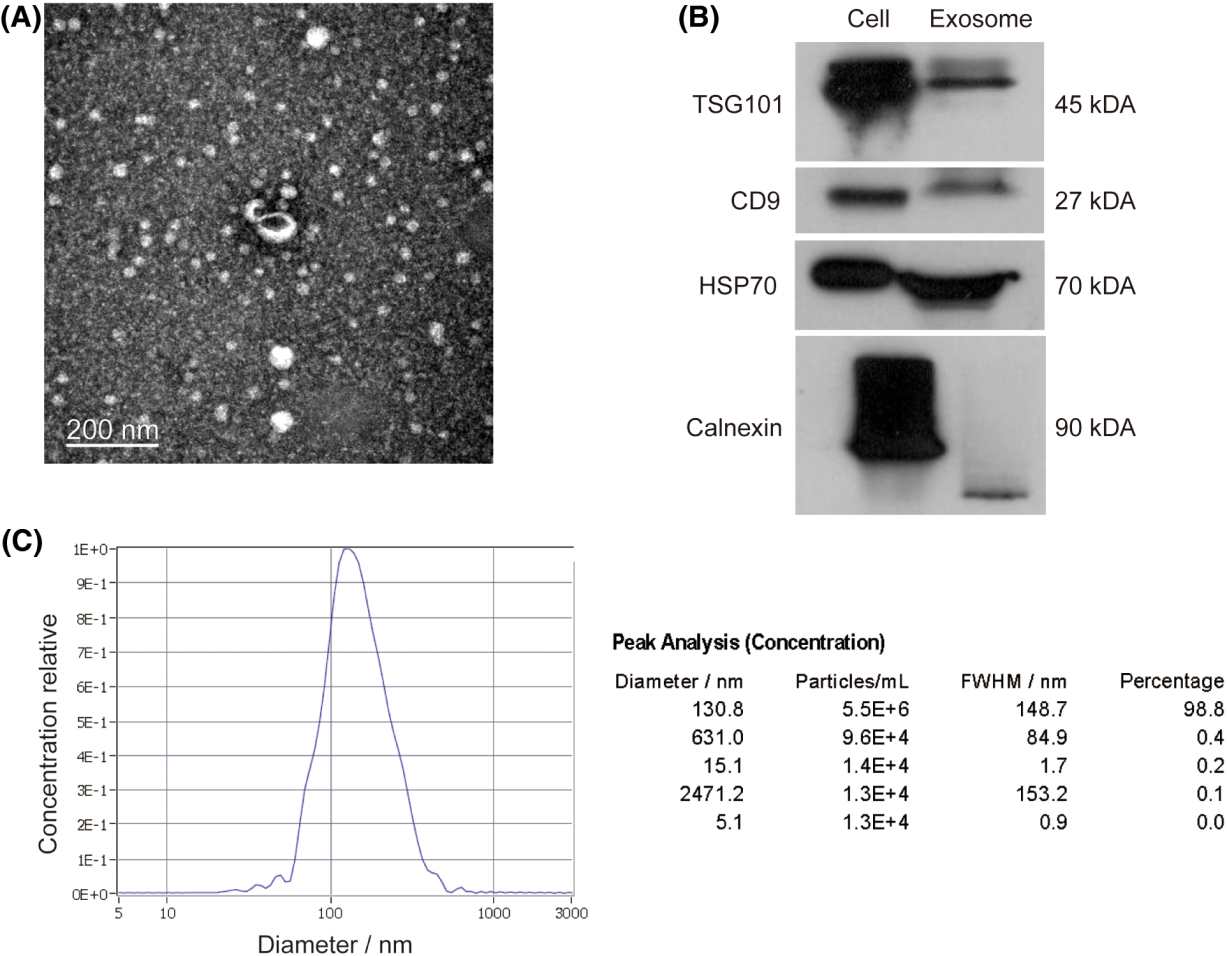
Identification of exosomes. (A) Exosomes extracted from plasma were identified by TEM. Magnification: ×10,000. Scale bar: 200 nm. Arrow indicates an exosome. (B) Western blot analysis of TSG101, CD63, and calnexin expression in exosomes and cell lysis solution. (C) The particle size detected by NTA.

TEM, NTA, and western blot analysis were used to characterize the isolated exosomes. The size and cup‐shaped morphology of exosomes were observed by TEM (JEOL‐JEM1400; Figure 1A). The exosomal markers TSG101, HSP70, and CD9 were enriched while calnexin was not expressed in the exosomal samples (Figure 1B, Figure S5). NTA tracked 1503 particles at a concentration of 2.2 × 10^10^/mL, and the mean exosomal diameter was consistent with the TEM results (Figure 1C).

### 3.2 Differential expression profile of circRNAs and mRNAs in the plasma exosomes of PD patients

**FIGURE 2:**
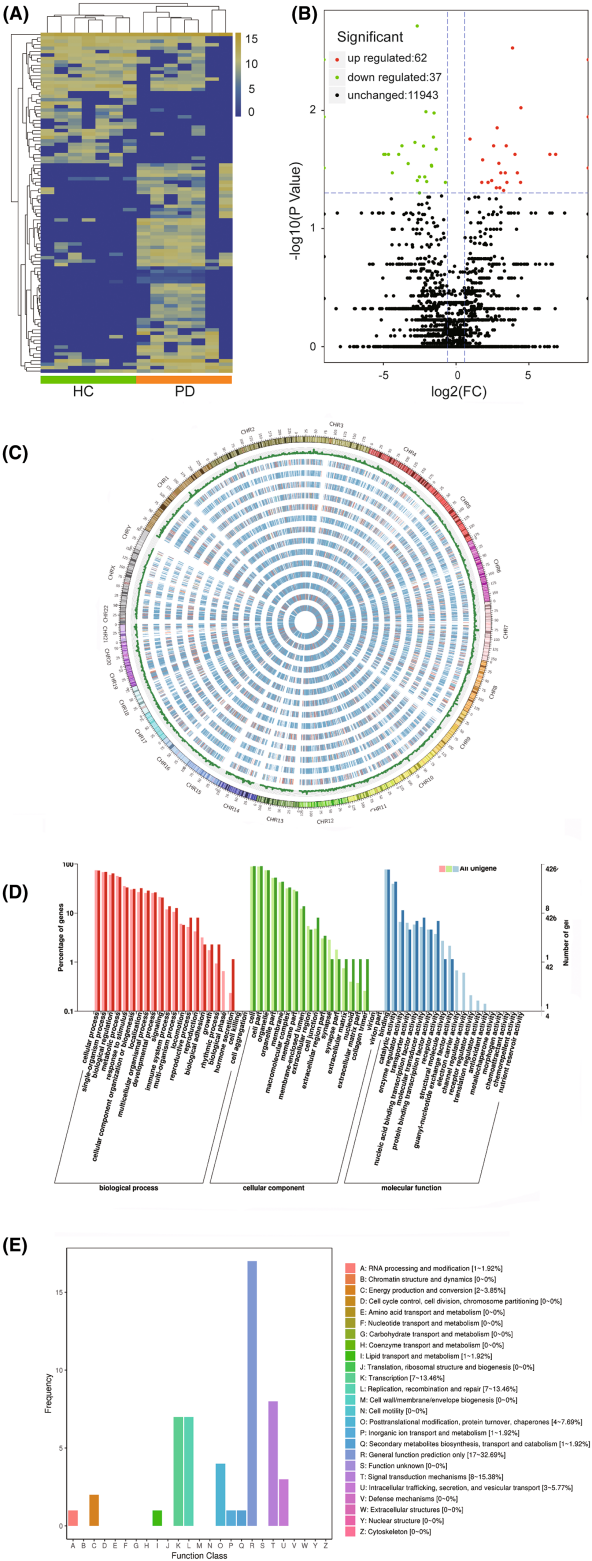
Expression of circular RNAs (circRNAs) and functional annotation of differentially‐expressed circRNAs (DEcircRNAs). (A) Hierarchical clustering of circRNAs in Parkinson's disease (PD) patients compared to healthy controls determined by NGS. The expression values are represented on a color scale. The intensity increases from blue (relatively lower expression) to yellow (relatively higher expression). Different columns represent different samples (*n* = 14), and each row represents a single circRNA. (B) Volcano plot of circRNAs in PD patients compared to healthy controls determined by NGS. Each point represents one single circRNA. The vertical dotted lines correspond to 1.5‐fold up‐ and downregulation (log_2_ scaled), and the horizontal dotted line represents a *p*‐value of 0.05. The red points indicate relatively higher expression, green points indicate relatively lower expression, and black points represent nondifferentially expressed genes. (C) Distribution of DEcircRNAs in chromosomes. The outer ring is the chromosome of the reference genome. The bar chart indicates the number of circRNAs in each location, and the heat chart indicates the expression of each sample. (D) GO classification of the host genes of DEcircRNAs. The abscissa is the GO classification, the left coordinate is the percentage of the number of host genes, and the right coordinate is the number of genes. (E) Clusters of orthologous groups of proteins (COG) function classification of the host genes of DEcircRNAs. The abscissa is the COG classification content, and the coordinate is the number of host genes.

NGS was performed on RNA obtained from the plasma exosomes of seven PD patients and seven healthy controls. NGS identified 9167 known and 2875 new circRNAs. The percentage of circRNAs mapped to the reads of each sample ranged from 80.75% to 95.12%. Depending on the position in the genome, circRNAs can be classified into three types: exonic, intronic, and intergenic. The proportions of each type of circRNA in each sample are shown in Figure S1. The circRNA distribution on the chromosome and the circRNA length distribution are shown in Figures S2 and S3, respectively. We found 62 upregulated and 37 downregulated circRNAs (Table S2), as well as the previously reported 160 upregulated and 377 downregulated mRNAs in PD patients compared with healthy controls.^35^ Hierarchical clustering analysis was performed on the 99 DEcircRNAs (Figure 2A) and 537 DEmRNAs, and the volcano plot in Figure 2B shows the variance among DEcircRNAs. The distribution of dysregulated circRNAs in chromosomes is shown in Figure 2C.

### 3.3 Functional annotation of DEcircRNAs

Of the identified 99 DEcircRNAs, 87 were assigned GO terms and 56 were assigned KEGG terms. In terms of DEmRNAs, 2362 target genes were GO annotated and 1521 were KEGG annotated. In terms of biological processes, cellular (GO:0009987) and single‐organism (GO:0044699) processes were the most significant categories. With respect to cellular components, cell (GO:0005623) and cell part (GO:0044464) were the most represented GO terms while binding (GO:0005488) and catalytic activity (GO:0003824) were the principal categories observed in the cellular component (Figure 2D). The KEGG term “ko04520 adherens junction” was significantly enriched in the KEGG pathway enrichment analysis. COG function classification identified “signal transduction mechanisms (8%–15.38%),” “transcription (7%–13.46%),” and “replication, recombination and repair (7%–13.46%)” pathways (Figure 2E).

### 3.4 The qPCR validation confirmed the downregulation of hsa‐SCMH1_0001 in PD patients

**FIGURE 3:**
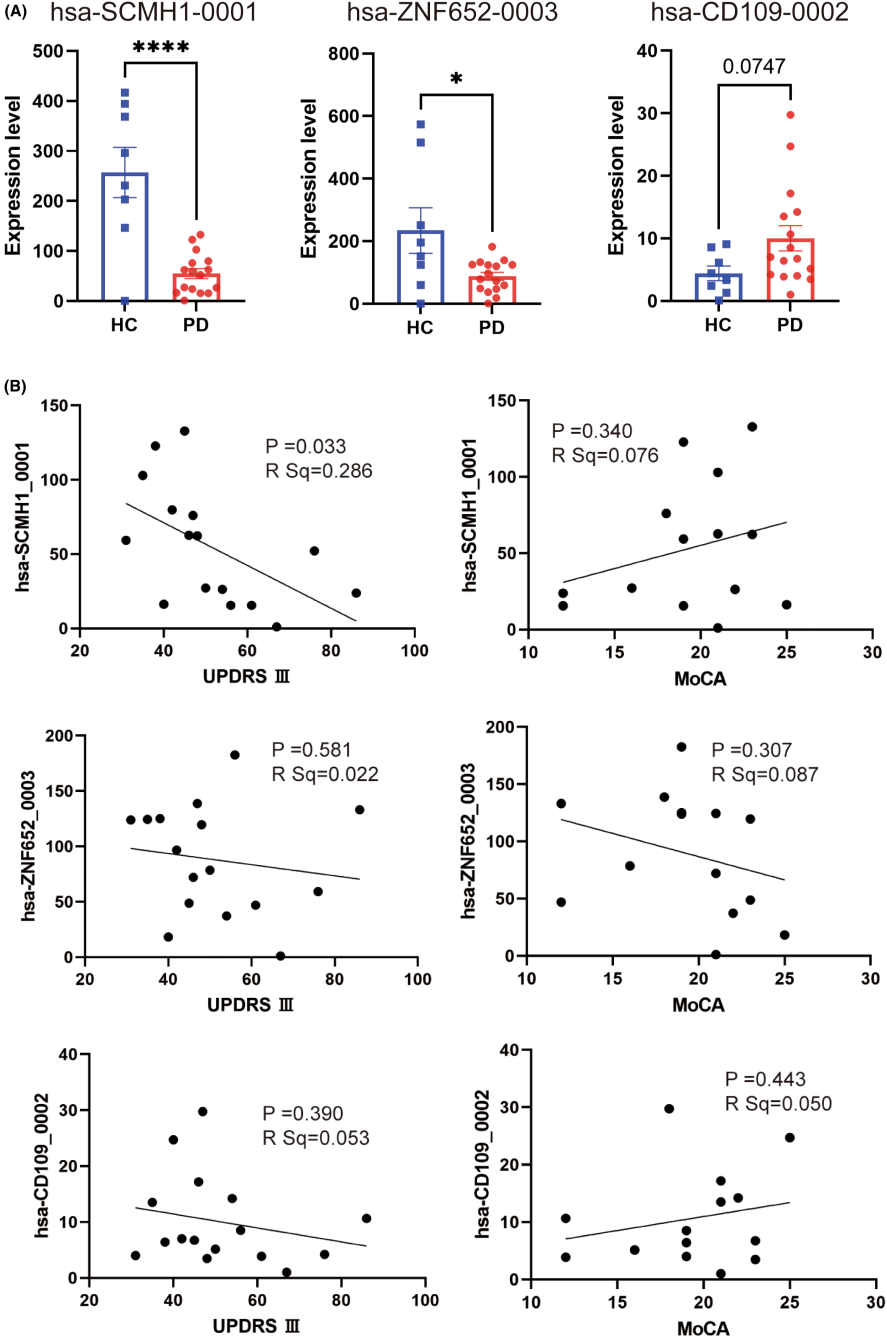
Circular RNAs (CircRNA) expression and clinical correlation analysis determined by qPCR. GAPDH was used as a housekeeping gene. (A) There was no significant difference in hsa‐CD109_0002 and hsa‐ZNF652_0003 expression between the two groups. hsa‐SCMH1_0001 was downregulated in PD patients. (B) hsa‐SCMH1_0001 expression was negatively correlated with the MDS‐UPDRS III score. There was no correlation between the hsa‐SCMH1_0001 expression level and MoCA score. hsa‐CD109_0002 and hsa‐ZNF652_0003 were not significantly associated with either the MDS‐UPDRS III or MoCA scores (*n* = 16) compared with healthy controls (*n* = 8; *p* < 0.05).

Based on the differential expression levels between the PD and healthy control groups and the functional annotation of the identified genes, we selected hsa‐CD109_0002, hsa‐SCMH1_0001, and hsa‐ZNF652_0003 for subsequent studies. The qPCR analysis revealed no significant differences in hsa‐CD109_0002 and hsa‐ZNF652_0003 expression levels between PD patients and healthy controls. hsa‐SCMH1_0001 expression levels were significantly downregulated (*p* < 0.05), consistent with the NGS data (Figure 3A).

### 3.5 hsa‐SCMH1_0001 was negatively correlated with the MDS‐UPDRS III

Correlation analysis was conducted between the expression levels of the two validated circRNAs and the clinical characteristics of PD patients including the MDS‐UPDRS III score and the Montreal cognitive assessment (MoCA). MDS‐UPDRS is the gold standard for the evaluation of PD^41^ while MoCA is a global cognition assessment tool used for the detection of MCI or dementia in PD.^32^ Correlation analysis demonstrated that hsa‐SCMH1_0001 expression level was negatively correlated with the MDS‐UPDRS III score such that patients with more severe motor symptoms expressed lower levels of hsa‐SCMH1_0001. There was no correlation between the expression level and MoCA score. In addition, hsa‐CD109_0002 and hsa‐ZNF652_0003 were not significantly associated with either of these scores (Figure 3B).

### 3.6 CircRNA–miRNA–mRNA interaction network of hsa‐SCMH1_0001

**FIGURE 4:**
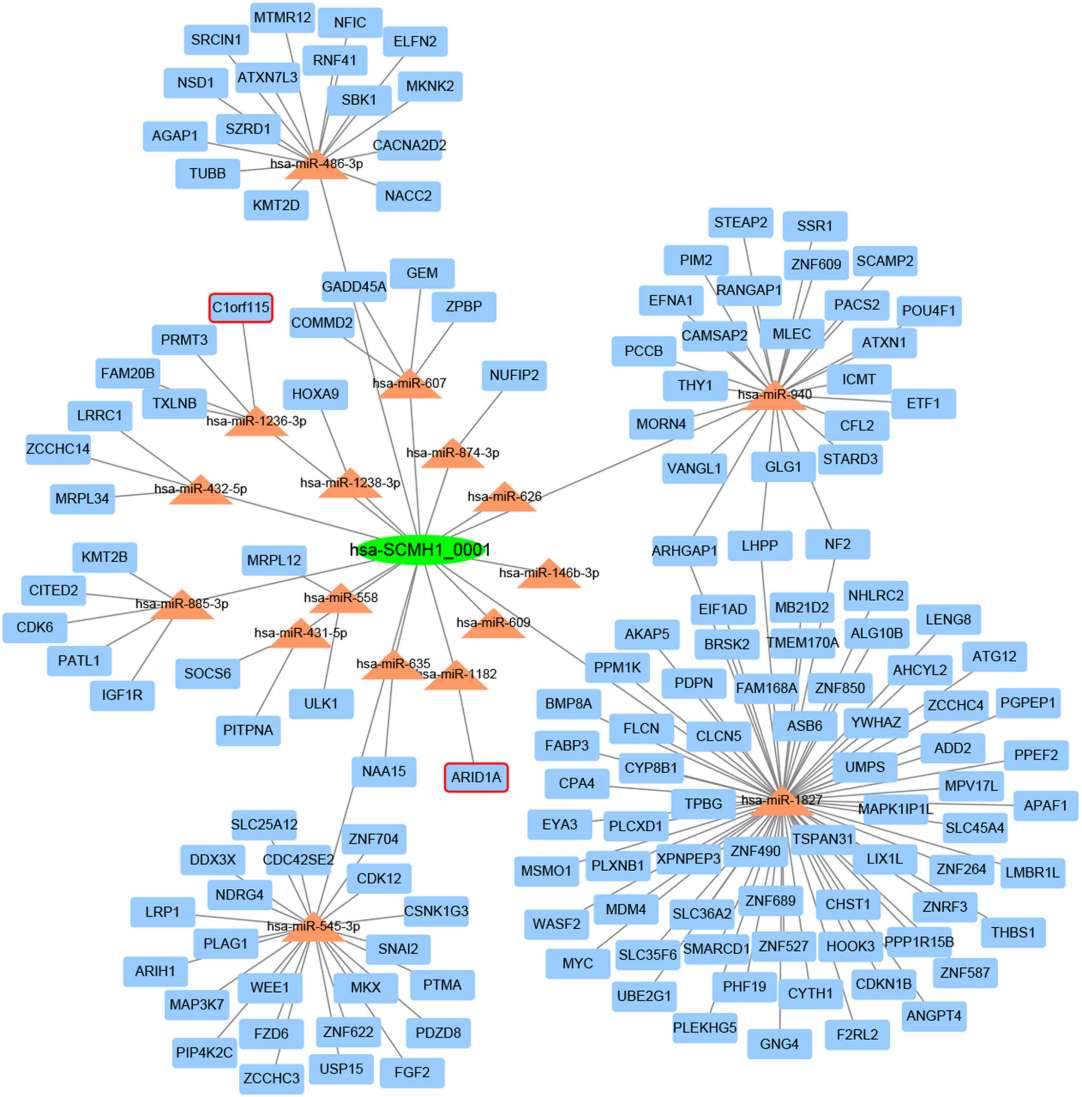
The hsa‐SCMH1_0001‐centered ceRNA network. The binding sites of circRNAs and miRNAs were predicted using CircInteractome. The target genes of miRNAs were predicted using TargetScanHuman, miRDB, and miRTarBase. Ellipse: circRNAs, triangle: miRNA, oval: mRNA. The mRNAs circled in red are DEmRNAs.

Based on the predicted binding miRNA partners and downstream target genes, we generated a ceRNA network using hsa‐SCMH1_0001 as the key circRNA (Figure 4). Seventeen miRNAs that have the potential to bind to hsa‐SCMH1_0001 were predicted through CircInteractome (Table 3). The miRNAs function by repressing mRNA translation or causing mRNA degradation. Using TargetScanHuman, miRDB, and miRTarBase we identified a total of 149 potential target genes. Target genes ARID1A and C1orf115 also belong to DEmRNAs obtained by sequencing. The circular structure of hsa‐SCMH1_0001 was analyzed using CircView (http://gb.whu.edu.cn/CircView/; Figure S4).^42^

**TABLE 3:** MiRNAs might bind to hsa‐SCMH1_0001.

miRNA	Site type	Context+ score
hsa‐miR‐1182	8mer‐1a	−0.39
hsa‐miR‐1236‐3p	7mer‐1a	−0.06
hsa‐miR‐1238‐3p	7mer‐1a	−0.081
hsa‐miR‐146b‐3p	7mer‐1a	−0.182
hsa‐miR‐431‐5p	7mer‐1a	−0.083
hsa‐miR‐432‐5p	7mer‐1a	−0.065
hsa‐miR‐486‐3p	8mer‐1a	−0.446
hsa‐miR‐545‐3p	8mer‐1a	−0.17
hsa‐miR‐558	7mer‐1a	−0.089
hsa‐miR‐607	7mer‐1a	0.043
hsa‐miR‐609	7mer‐1a	−0.13
hsa‐miR‐626	7mer‐8a	−0.221
hsa‐miR‐635	7mer‐1a	−0.083
hsa‐miR‐1827	8mer‐1a	−0.299
hsa‐miR‐874‐3p	7mer‐8a	−0.185
hsa‐miR‐885‐3p	7mer‐1a	−0.175
hsa‐miR‐940	7mer‐8a	−0.216

## 4 DISCUSSION

**FIGURE 5:**
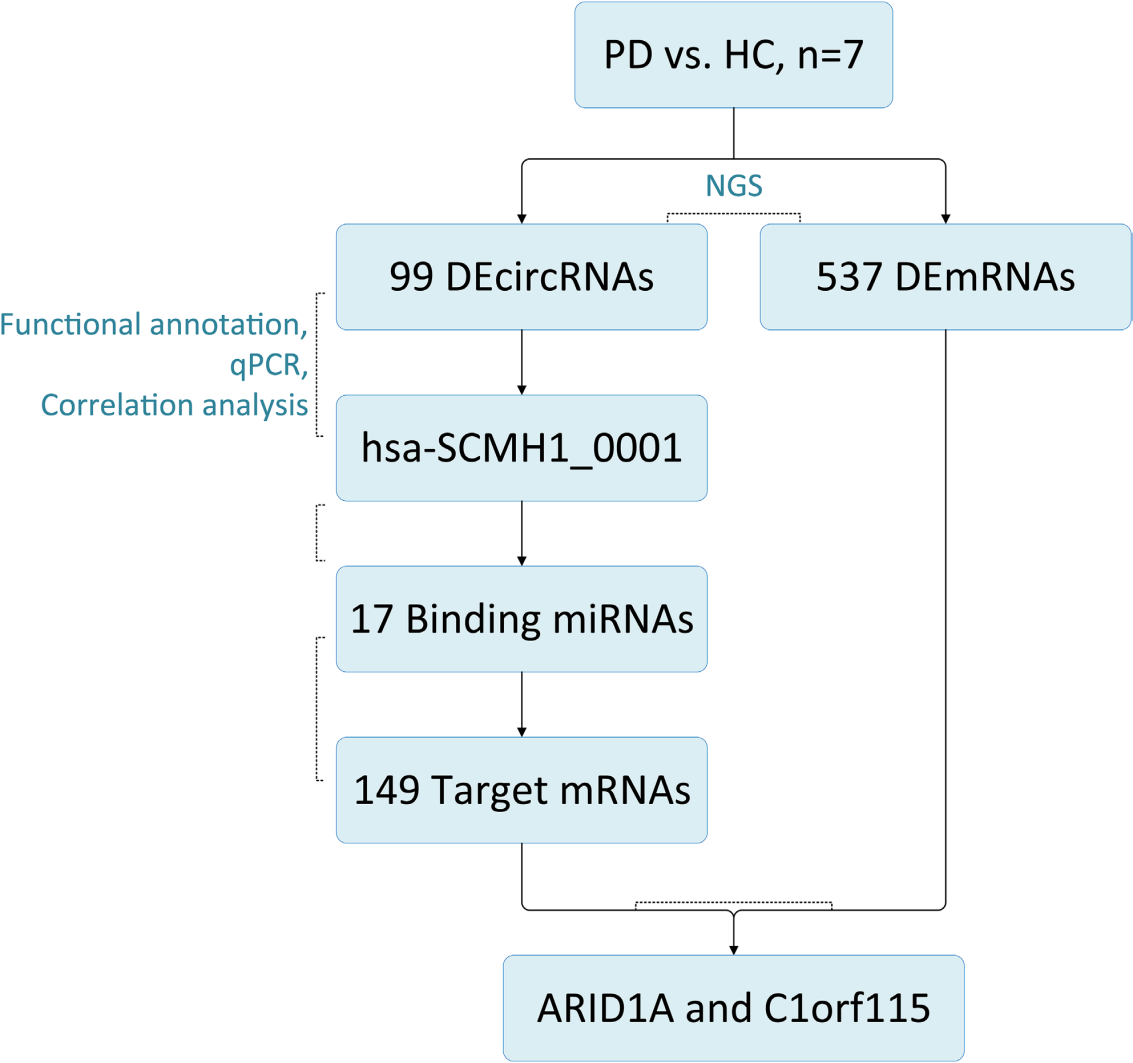
Flowchart of the study. NGS was performed on seven PD patients and seven healthy controls, and a total of 99 DEcircRNAs and 537 DEmRNAs were identified. Through functional annotation, qPCR verification, and correlation analysis with clinical features, hsa‐SCMH1_0001 was found to be associated with PD. Seventeen binding miRNAs were predicted by CircInteractome, and the target mRNAs predicted by TargetScanHuman, miRDB and miRTarBase were intersected to obtain 149 mRNAs. This result was intersected with the DEmRNAs identified by sequencing, and ARID1A and C1orf115 were screened out.

In this study, we successfully isolated and identified exosomes from human plasma. We examined the circRNA and mRNA expression profiles in peripheral exosomes from PD patients and healthy controls using NGS, functional annotation, and qPCR, and identified DEcircRNAs and DEmRNAs. By analyzing the correlation between the expression levels of circRNAs of interest and the MDS‐UPDRS III scores in PD patients, we were able to identify hsa‐SCMH1_0001 as a circRNA with strong clinical relevance. Based on our ceRNA network, we identified 17 potential binding miRNAs with 149 potential target genes. By comparing these predicted target genes with the previously reported DEmRNAs, we identified two genes of interest, ARID1A and C1orf115. Figure 5 illustrates the flow of the entire study.

The patients enrolled in this study were all advanced PD patients with typical motor symptoms, who had been taking anti‐PD drugs regularly for at least 6 years. Considering the heterogeneity of the clinical patients, we adopted strict inclusion and exclusion criteria in the selection of NGS subjects, with a strict limitation on the age and sex ratios between the two groups.

Although brain tissue is undoubtedly the best sample for studying neurodegenerative diseases, it is impossible to obtain high‐quality brain tissue samples from living patients. Therefore, we decided to study the transcriptome expression in plasma exosomes since they carry genetic information from the central nervous system across the BBB. This approach has been widely used in the study of miRNAs in neurodegenerative diseases, especially Alzheimer's disease.^33^ In terms of PD, studies have shown that α‐synuclein, L1CAM, DJ‐1, Tau, and lnc‐RNA‐POU3F3, as well as various miRNAs, including miR‐1, miR‐19b‐3p, miR‐10a‐5p, miR‐153, miR‐409‐3p, miR‐331‐5p, miR‐505, miR‐24, and miR‐195, can be used as potential biomarkers.^43^ Previously, we have shown that lnc‐MKRN2‐42:1 in plasma exosomes may be involved in the occurrence of PD.^35^

CircRNAs are highly abundant in the mammalian brain and enriched in synaptoneurosomes. Neuronal circRNAs are upregulated during neuronal differentiation and have significantly conserved sequences and expression patterns.^13^ Thus, circRNAs could be promising biomarkers in PD. The concept of ceRNA was first proposed by Salmena et al.^44^ to explain the interactions between RNAs. CircRNA ciRS‐7/CDR1as that acts as a sponge for miR‐7 is thought to be associated with PD.^45^ In addition, Kumar et al.^46^ demonstrated that circRNA circzip‐2 may act as sponge for miR‐60, which has an important role in various processes associated with PD. A study by Ravanidis et al.^47^ also identified six circRNAs that were significantly downregulated in PD patients, four of which could distinguish PD patients from healthy control subjects.

The host gene of hsa‐SCMH1_0001 is the Scm Polycomb Group Protein Homolog 1 (SCMH1) gene, located on human chromosome 1. Exosomal circRNA SCMH1 was previously shown to be decreased in the plasma of patients with acute ischemic stroke. In addition, circRNA SCMH1 could significantly improve the functional recovery of stroke animal models by enhancing neuroplasticity, and inhibiting glial responsiveness and peripheral immune cell infiltration.^48^ In our study, we found that plasma exosomal hsa‐SCMH1_0001 was significantly downregulated in PD patients compared to healthy controls. Our findings are consistent with the expression trend observed in stroke patients, suggesting that hsa‐SCMH1_0001 may play an a role in some central nervous system diseases.

Interestingly, among the DEcircRNAs, hSA‐SCMH1_0001 expression was negatively correlated with MDS‐UPDRS III, suggesting that hSA‐SCMH1_0001 may be a potential biomarker for evaluating PD severity. However, this hypothesis remains to be confirmed. Fischer et al.^49^ suggested that the levodopa monotherapy strategy in PD patients was associated with worse disease outcomes in BDNF rs6265 T carriers while L1CAM exosomal Linc‐POU3F3, L1CAM exosomal α‐syn, and GCase activity could be used to assess the severity of PD.^50^ PD is a neurological disease with complex clinical manifestations that can be accompanied by a variety of non‐motor symptoms. Ahamadi et al.^51^ found that the rate of progression based on MDS‐UPDRS I was approximately 60% lower in carriers of LRRK2 gene mutations compared to noncarriers, suggesting that the progression of nonmotor symptoms in PD could be predicted by LRRK2 gene mutations. Using machine learning, Ramezani et al.^52^ found that SNCA genes were related to the cognitive ability of idiopathic PD patients. In our study, correlations between the MoCA score and DEcircRNA expression were not significant. This may be due to the relatively small amount of circRNA extracted from exosomes, which leads to the identification of fewer DEcircRNAs and fewer KEGG functional pathway enrichment results. Taken together, our study showed that hsa‐SCMH1_0001 was significantly downregulated in PD patients, and that decreased expression levels were associated with aggravation of motor symptoms. Therefore, we speculated that the downregulation of hsa‐SCMH1_0001 may be involved in the pathogenesis of PD and may reflect the occurrence of PD.

Based on the ceRNA hypothesis, the expression of circRNA should be positively correlated with the expression of target genes. In our study, hsa‐SCMH1_0001 was positively correlated with ARID1A and C1orf115, suggesting that hsa‐SCMH1_0001 may participated in the occurrence of PD through the regulation of ARID1A and C1orf115. We did not compare the predicted circRNA‐binding miRNAs with the DEmiRNAs obtained by sequencing because in theory, the DEcircRNAs do not necessarily lead to a large number of changes in the expression of specific miRNAs. In order to predict target genes more reliably, we used different algorithms of multiple databases.

Our work provided a novel insight into the molecular mechanism of PD. However, more studies, both in vitro and in vivo, are required to elucidate the function of hsa‐SCMH1_0001 and its target genes in PD. Furthermore, in order to realize its value as a potential biomarker, future studies should use a larger sample size for clinical feature prediction, as well as relax the inclusion criteria and include patients with different disease severity, especially early PD patients.

## 5 CONCLUSIONS

In conclusion, this study identified hsa‐SCMH;1_0001 as a PD‐associated circRNA in the plasma exosomes of PD patients. Correlation analysis with MDS‐UPDRS III and ceRNA analysis revealed possible molecular mechanisms of action of hsa‐SCMH1_0001 through ARID1A and C1orf115. Our findings furthered the understanding of the pathogenesis of PD and provided a potential tool for clinical diagnosis.

## AUTHOR CONTRIBUTIONS

Qiao Wang and Huizhi Wang and Xin Zhang: data curation; Qiao Wang and Zhibao Li: formal analysis; Fangang Meng, Yilei Xiao, and Guoen Cai: funding acquisition; Qiao Wang, and Chong Liu: investigation; Qiao Wang, and Chunlei Han: methodology; Fangang Meng and Chunlei Han: project administration; Tingting Du and Xin Zhang: resources; Jianguo Zhang and Fangang Meng: supervision; Tingting Du: validation and Yilei Xiao: visualization; Qiao Wang: visualization; Qiao Wang: writing‐original draft; Qiao Wang: writing‐review and editing. Chunlei Han, Tingting Du, Qiao Wang, and Huizhi Wang, and Xin Zhang was a major contributor in writing the manuscript. All authors read and approved the final manuscript.

## FUNDING INFORMATION

This research was funded by Beijing Natural Science Foundation Program and Scientific Research Key Program of Beijing Municipal Commission of Education (grant number: KZ201910025036), National Natural Science Foundation of China (grant numbers: 81971070, 81901314), the Natural Science Foundation of Shandong Province of China (grant number: ZR2021MH303), and the Taishan Scholar Project of Shandong Province of China (grant number: tsqn202103200).

## CONFLICT OF INTEREST STATEMENT

The authors declare that they have no competing interests.

## Supporting information


Table S1.

Figure S1.

## Data Availability

The datasets generated and/or analyzed during the current study are not publicly available due we have unpublished studies from this data but are available from the corresponding author on reasonable request.
