# Supplementary material for: Transcriptome sequencing of circular RNA reveals the involvement of hsa‐SCMH1_0001 in the pathogenesis of Parkinson's disease
Source: CNS Neurosci Ther. 2023 Sep 4;30(3):e14435. doi: 10.1111/cns.14435 (PMC10916443; doi:10.1111/cns.14435)
Supplement: Supplementary file 2 — Figure S1. [file CNS-30-e14435-s001.pdf]

Uncropped gels (Figure 1B).

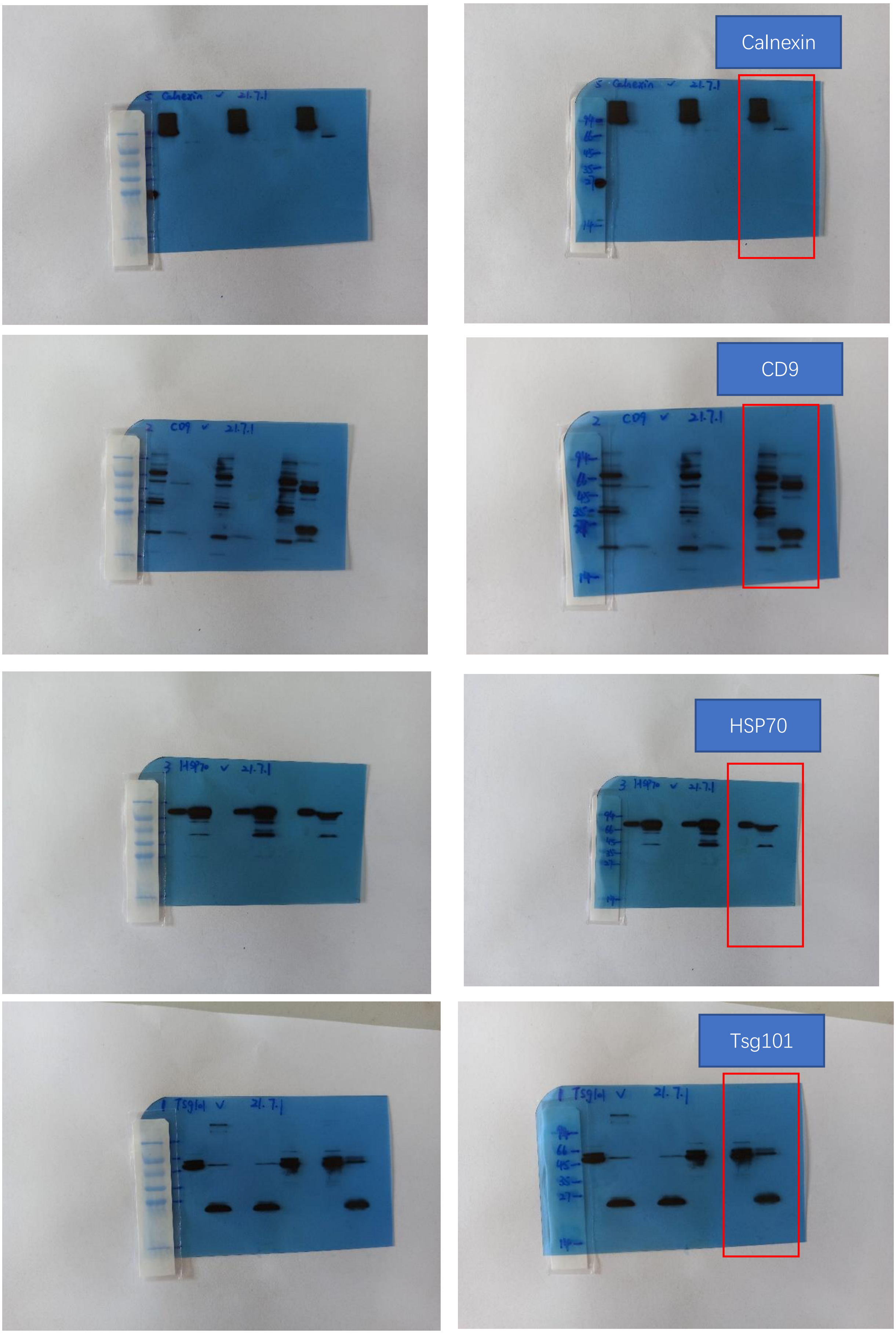

The uncropped images of the gels contain the experimental results of other researchers. The samples involved in this study are the last two samples of each original picture.

The experiments of western blotting have been performed many times in our group (1). The antibodies against Tsg101, CD9, HSP70, and Calnexin were purchased from Proteintech and abcam, respectively. In line with pervious references, the reports also exploited those antibodies to detect the protein of interest and some of them with several nonspecific blots (2-4), as following:

1. Wang Q, Han CL, Wang KL, Sui YP, Li ZB, Chen N, Fan SY, Shimabukuro M, Wang F, Meng FG. Integrated analysis of exosomal lncRNA and mRNA expression profiles reveals the involvement of lnc-MKRN2-42:1 in the pathogenesis of Parkinson's disease. CNS Neurosci Ther. 2020 May;26(5):527-537. doi: 10.1111/cns.13277. Epub 2019 Dec 8. PMID: 31814304; PMCID: PMC7163584
2. Wang ZG, He ZY, Liang S, Yang Q, Cheng P, Chen AM. Comprehensive proteomic analysis of exosomes derived from human bone marrow, adipose tissue, and umbilical cord mesenchymal stem cells. Stem Cell Res Ther. 2020 Nov 27;11(1):511. doi: 10.1186/s13287-020-02032-8. PMID: 33246507; PMCID: PMC7694919.
3. Guan D, Li Y, Cui Y, Guo Y, Dong N, Li G, Dai Y, Ji L. Down-regulated miR-374c and Hsp70 promote Th17 cell differentiation by inducing Fas expression in experimental autoimmune encephalomyelitis. Int J Biol Macromol. 2020 Jul 1;154:1158-1165. doi: 10.1016/j.ijbiomac.2019.11.147. Epub 2019 Nov 19. Erratum in: Int J Biol Macromol. 2021 Jan 1;166:1617-1618. PMID: 31756487.
4. Deng Q, Fang Q, Xie B, Sun H, Bao Y, Zhou S. Exosomal long non-coding RNA MSTRG.292666.16 is associated with osimertinib (AZD9291) resistance in non-small cell lung cancer. Aging (Albany NY). 2020 May 6;12(9):8001-8015. doi: 10.18632/aging.103119. Epub 2020 May 6. PMID: 32375124; PMCID: PMC7244069.
